# A Forward Dynamic Modelling Investigation of Cause-and-Effect Relationships in Single Support Phase of Human Walking

**DOI:** 10.1155/2015/383705

**Published:** 2015-06-14

**Authors:** Michael McGrath, David Howard, Richard Baker

**Affiliations:** ^1^School of Health Sciences, University of Salford, Salford M6 6PU, UK; ^2^School of Computing, Science and Engineering, University of Salford, Salford M5 4WT, UK

## Abstract

Mathematical gait models often fall into one of two categories: simple and complex. There is a large leap in complexity between model types, meaning the effects of individual gait mechanisms get overlooked. This study investigated the cause-and-effect relationships between gait mechanisms and resulting kinematics and kinetics, using a sequence of mathematical models of increasing complexity. The focus was on sagittal plane and single support only. Starting with an inverted pendulum (IP), extended to include a HAT (head-arms-trunk) segment and an actuated hip moment, further complexities were added one-by-one. These were a knee joint, an ankle joint with a static foot, heel rise, and finally a swing leg. The presence of a knee joint and an ankle moment (during foot flat) were shown to largely influence the initial peak in the vertical GRF curve. The second peak in this curve was achieved through a combination of heel rise and the presence of a swing leg. Heel rise was also shown to reduce errors in the horizontal GRF prediction in the second half of single support. The swing leg is important for centre-of-mass (CM) deceleration in late single support. These findings provide evidence for the specific effects of each gait mechanism.

## 1. Introduction

A comprehensive biomechanical understanding of human walking should be the foundation of a range of fields including rehabilitation, prosthetics, and robotics. Unfortunately no such understanding exists. For many years the most widely accepted was “*The Determinants of Gait*” [1]. This started with an extremely simple model of walking (compass gait) and added complexity sequentially through six kinematic gait mechanisms (pelvic rotation, pelvic obliquity, knee flexion, lateral displacement of the CM, and knee and ankle mechanisms), which were presented as progressively reducing energy consumption by translating the CM “*through a sinusoidal pathway of low amplitude in which the deflections are gradual*.” Whilst the overall approach is persuasive and provides an attractive framework within which to teach, experimental studies [2–7] have largely discredited the approach. The problem would appear to be that attractive conceptual models were never modelled mathematically or subjected to experimental validation.

A number of simple mathematical models of walking have been described. The simplest of these is the inverted pendulum (IP) [8–14] which models the mass of the body as a single point at the end of a straight, massless, and rigid “*leg*.” This models elements of single support remarkably well but cannot be applied to double support and cannot track the characteristic “double bump” of the vertical component of the ground reaction force [15]. The Spring Loaded Inverted Pendulum (SLIP) model [16–21] which incorporates a spring controlled telescopic component in the leg can produce a realistic centre-of-mass (CM) motion and hence better GRF curves. It clearly does not reflect human anatomy, however, and thus cannot give insight into how joint angle or moment time-histories coordinate during walking.

The advent of more powerful and readily available computer processors has led to substantial advances in the sophistication of forward dynamic models of walking which aim to model human anatomy and physiology in considerable detail in two or three dimensions [8, 22, 23]. A number of techniques such as induced acceleration analysis [23, 24] and decomposition of the ground reaction force [8] have been developed to analyse the outputs of such models. The very complexity of these models, however, precludes the sort of sequential development from simple to more complex models which was the key pedagogic strength of the* Determinants of Gait* approach. Some researchers have begun to highlight the benefit of “*intermediately complex*” models [25] but further exploration is needed.

A particularly important issue is that studies of a single model can only be descriptive. Previous papers which have presented a decomposition of the ground reaction [8, 26–28], for example, are essentially descriptive of how we walk but give little insight into why we do so in a particular fashion or what mechanisms are required to achieve this. Developing a series of models with different attributes and comparing the results is a more appropriate way of addressing such questions.

The aim of this paper is to adopt the overall approach of the* Determinants of Gait* in presenting a series of models of single support during human walking of increasing complexity but using rigorous forward dynamics techniques similar to those used in the current generation of more sophisticated models. Through this the contributions to walking of specific gait mechanisms such as knee flexion and heel lift will be investigated.

## 2. Method

**Figure 1:**
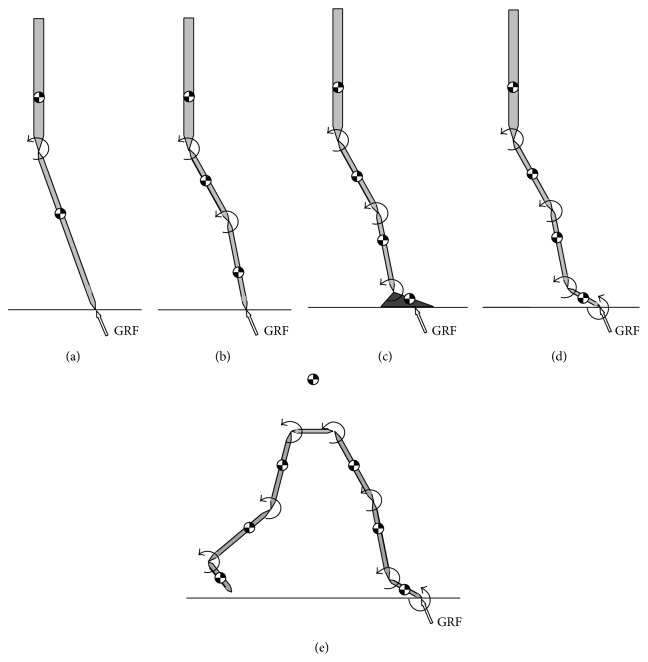
Free body diagrams of the five different walking models: (a) Model 1 advances an IP model by incorporating a HAT segment; (b) Model 2 adds a knee joint; (c) Model 3 adds a static foot and an ankle moment; (d) Model 4 allows the foot segment to move; (e) Model 5 adds a swing leg.

A sequence of five 2D sagittal plane joint actuator driven models was designed (see Figure 1), each incorporating an additional degree of freedom (DOF) or actuator than the previous one. Each was used in a forward dynamic simulation of single support to track the same 2D joint kinematic data [29] with an assumption that the trunk segment remains vertical throughout single support [30–33]. Winter's data represent 19 healthy young adults walking at natural cadence. They were captured with a CCD video camera at 60 Hz. The position, in the sagittal plane, of the markers that were placed over the joint centres, was digitised to within an estimated accuracy of 1 mm. They were then filtered with a fourth-order, zero-lag Butterworth filter, with a cut-off of 6 Hz [34]. They are particularly appropriate for the current study as they are pure sagittal plane measurements, with a trunk defined by an essentially vertical axis (rather than more recent systems which introduce an offset due to the relative height of anterior and posterior superior iliac spines). Their pedigree is well established in the literature and data for both joint and segmental kinematics is widely available [29].

Lagrangian dynamics were used to derive the equations of motion describing the applied moments (*M*_*i*_) as a linear function of the generalised accelerations (${\ddot{q}}_{i}$) with functions of the state vector (*q*_*i*_, ${\dot{q}}_{i}$) as coefficients. These were written in matrix form and inverted to obtain the generalised accelerations which were then integrated numerically using a Taylor expansion method. Vertical and horizontal components of the GRF were calculated from the linear and angular segmental accelerations. This is an extension of the method first presented by McGrath et al. [15] which includes an electronic appendix detailing the derivation of the equations used to calculate these quantities. Each joint moment was defined by 21 equally spaced nodes with piecewise cubic polynomial interpolation for intermediate values [35].

In each of the simulation initial angular positions (*q*_*i*_) and velocities (${\dot{q}}_{i}$), the nodal values for all joint moments were optimised to minimise the root mean square difference between the model joint angles and velocities and the reference kinematic data. A penalty was incorporated into the cost function of models with a knee in order to prevent knee hyperextension. Initial values for all the optimised variables were taken from Winter's data. A global optimisation algorithm [36] was used and the result was then taken as the input to a local optimisation function [37], in order to produce the most accurate solution.

To ensure a fair comparison between the different models, the gait parameters used to perform simulations were the same for each model. An average walking velocity of 1.2 m/s was taken from Winter's data [29] and a single gait cycle was calculated to take approximately 0.9 seconds. Many sources cite a single support period as being approximately 40% of the full gait cycle [38–41] and so the time for which the simulations were run was 0.36 seconds.

**Figure 2:**
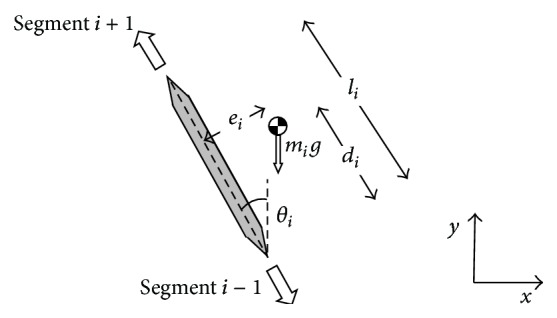
The geometry of any given segment.

In all of the models described below, the segment properties are defined in a similar way. Referring to Figure 2, the angular position of “*segment i*” was defined as the angle the segment* made with the vertical axis*. The right hand rule was used for angles, angular velocities, angular accelerations, and moments (i.e., anticlockwise was positive). The total length of the segment was *l*_*i*_. The position of the CM of the segment was defined by two values, *d*_*i*_ and *e*_*i*_, where *d*_*i*_ is parallel to the length of the segment and *e*_*i*_ is perpendicular. The values for mass (*m*), *d*, *e*, and moment of inertia (*I*) for the segments were assigned using Winter's formulae [29] for a person of 80 kg mass and 1.8 m height. The direction of progression is in the positive *x* direction and the positive *y* direction corresponds to the vertical direction.

### 2.1. Model 1: Inverted Pendulum with a HAT Segment

A two-DOF model was developed by adding a HAT segment to a traditional IP model (Figure 1(a)). No foot mechanism was used so the model pivots about a workless constraint at a point on the ground. An actuation moment was applied at the hip joint and adjusted by the optimiser to achieve the best kinematic match between simulation and experiment. As experimental data were required for the segment angle of the leg as a whole, against which to compare the simulation results, the angle of the single leg segment was calculated from coordinate data for the ankle and hip. These were derived from the experimental segment angle data.

### 2.2. Model 2: Introducing the Knee

A three-DOF model was developed by separating the leg segment of Model 1 into femur and shank/foot segments (Figure 1(b)). Again, no foot mechanism was used so the model pivots about a workless constraint at a point on the ground. Actuation moments were applied at the hip and knee joints and adjusted by the optimiser to achieve the best kinematic match.

### 2.3. Model 3: Adding a Static Foot

In the previous model, the GRF had acted at a single point, where the first segment met the ground, which is unrealistic as the centre of pressure (COP) moves forward during stance [41]. Therefore, an alternative three DOFs model was proposed that incorporated a static foot segment (i.e., always in the foot flat state) and an extra moment applied at the ankle joint (Figure 1(c)). This retained three DOFs but provided a better support mechanism and mass distribution. Again, the optimiser adjusted the joint moments to achieve the best kinematic match.

### 2.4. Model 4: Incorporating Heel Rise

The next model was the same as the previous one except that it allowed heel rise to occur, increasing the number of DOFs to four (Figure 1(d)). When the COP reached the anterior pivot (metatarsal break), the foot segment was free to rotate. Again, the optimiser adjusted the joint moments to achieve the best kinematic match.

### 2.5. Model 5: Adding a Swing Leg

In the final model, a swing leg was added to the previous model (Figure 1(e)). In this case, six joint moments were applied at the ankles, knees, and hips. Again, the optimiser adjusted the joint moments to achieve the best kinematic match.

## 3. Results

**Figure 3:**
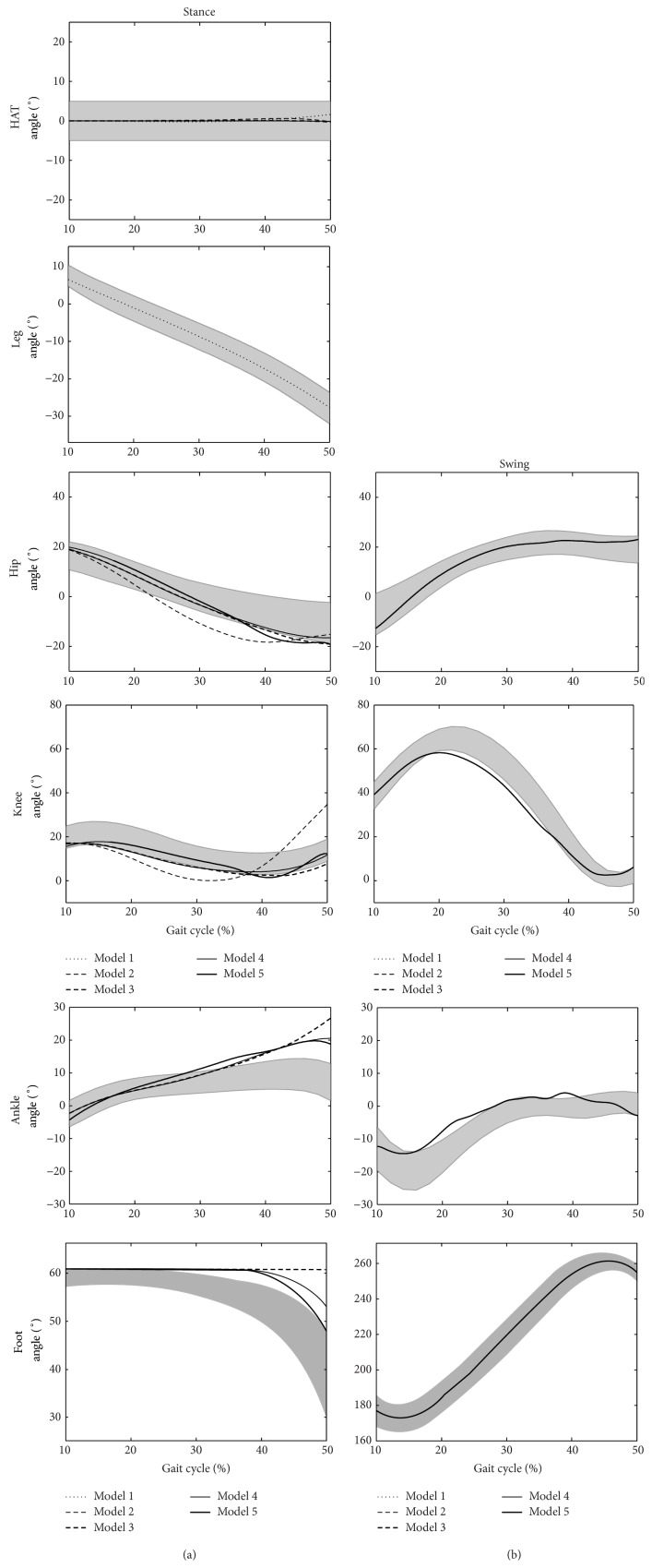
The joint and segment angle predictions for each model ((a) show the stance leg predictions and (b) show the swing leg predictions).

**Figure 4:**
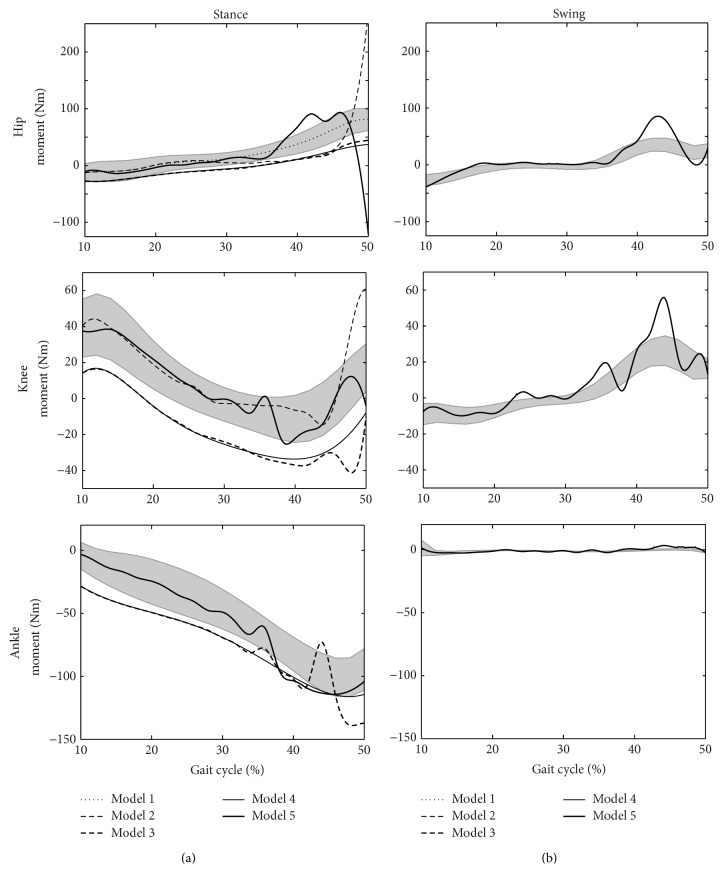
The joint moment predictions for each model ((a) show the stance leg predictions and (b) show the swing leg predictions).

**Figure 5:**
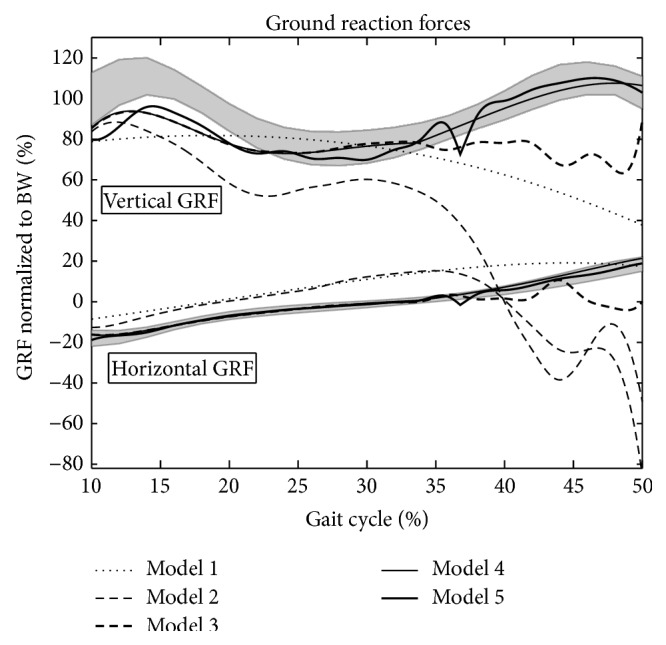
The predictions of the vertical and horizontal components of GRF for each model.

The simulation results are presented in Figures 3 to 5. The shaded areas on each of the plots show ±1 standard deviation from the experimental mean. The various lines show the simulation results using the different models. Anticlockwise moments are positive, according to the right hand rule for moments, as opposed to some gait analysis conventions.

Figure 3 shows segment and joint angle predictions for each of the models, compared to the experimental data. Referring to Table 1, Model 1 (IP and HAT) had very low RMS errors for both segments (<1°). For Model 2, the kinematic simulation results looked encouraging for the first half of single support but the leg, particularly the shank/foot segment, rotated too far forward by the end of single stance. Model 3 was able to achieve a result where all segment angles remained within ±1 standard deviation throughout single support. This was also the case for Model 4, apart from the foot segment which rose too slowly in the second half of stance. Model 5 was able to produce a very strong kinematic match with a mean segment angle RMS error of 1.4°. The stance foot was once again slow to rise in late stance but its final angular position was just on the edge of the desired range (1.07 standard deviations), without need for a targeted penalty function being added to the optimisation objective.

Figure 4 shows the joint moment predictions for each of the models, compared to the experimental data. The hip moments applied to Model 1 changed minimally from the initial curve (i.e., the experimental mean) with an RMS error of the order of 10^−5^ Nm. The predicted moment curves for Model 2 were within ±1 standard deviation of the experimental means during the first half of the simulation but not after that. The joint moment time-histories of Models 3 and 4 gave interesting results. Each curve was similar in shape to the experimental results but was translated outside its standard deviation range; the hip showed more extension moment, the knee more flexion moment, and the ankle more plantarflexion moment. The addition of a swing leg caused the moment curves of Model 5 to stay mostly within the experimental ranges for the first half of the simulation. However, there were a number of spikes in the curves in the second half (notably swing knee and both hips) but the general patterns exhibited were close to the experimental means.

Figure 5 shows the GRF component curves. Model 1 produced simulation results similar to those of previous IP models [8, 9, 13] but asymmetric about the pivot point because the final leg angle was greater than the initial leg angle. This meant the vertical GRF did not have either of the two peaks and dropped quickly in late single support. The horizontal GRF was too low in magnitude.

The vertical GRF for Model 2 showed the beginnings of an initial peak (88% bodyweight (BW) rather than 111% BW), dropping to a midstance trough. However, the predictions for both vertical and horizontal force components deteriorated considerably during the second half of the simulation. The vertical GRF curve of Model 3 clearly showed a distinct initial peak and midstance trough, although the peak was still not as high as the experimental one (94% BW rather than 111% BW). The horizontal GRF improved in the first half of stance, staying within the standard deviation range. Both curves strayed further from the experimental means in the second half of stance, although not quite as drastically as Model 2. For Model 4, the first peak of the vertical GRF component was once again lower than the experimental data measurements (94% BW) but this was the only time at which either vertical or horizontal values were outside the standard deviation range. For the first time, the second vertical GRF peak was present. Model 5 presented GRF curves that were similar to those of Model 4, except with a more obviously visible gradient change at the transition between the foot flat and heel rise phases of single support. Also, after heel rise, both curves were closer to the experimental means than their equivalents for Model 4.

## 4. Discussion

Model 1 was only slightly more complex than the conventional IP model [8, 9, 13, 15]. A HAT segment was added and a hip moment controlled the joint between the two segments. The kinematics and GRF curves were very similar to previous IP analyses [8, 9, 13]; however, the traditional IP models do not provide insight into the role of the hip joint moment. The low kinematic RMS error (Figure 3, mean value of 0.38°) was achieved with a moment curve that was practically the same as the experimental mean (Figure 4). This suggests that the role of the hip moment is to maintain a vertical HAT segment. This agrees with Arnold et al. [42] in early single support where the dominant contributor to hip acceleration is the gluteus maximus. It gives greater insight into the role of the hip extensors than decomposition of the ground reaction [8] which, whilst indicating that hip extensor activity is an important contributor to the ground reaction during this phase, gives little insight into the mechanism which requires this.

Model 2, which has a knee joint controlled by the optimiser determined knee moment, gives the least good match of any of the models in terms of kinematics (Figure 3) and particularly the ground reaction force (Figure 5). A particular feature is that the tibia appears to fall too far forwards in late stance. Model 3, which introduces a foot and ankle, shows a much better match for kinematics and the ground reaction force showing how the anterior movement of the ground reaction under the foot through single support is an important mechanism to ensure that the knee extensors are able to adequately control the joint. This helps explain previous observations [27, 28] that the plantarflexors are important contributors to the ground reaction in late single support.

Model 2 exhibits poor generation of a ground reaction force in late single support (Figure 5). This is partly restored by the incorporation of the foot and ankle in Model 3 but it is only Models 4 and 5, which allow heel rise, that show an ability to generate the second “bump” of the ground reaction. This is in agreement with the observation of Anderson and Pandy [8] that the plantarflexors are unable to make a significant contribution to the ground reaction until after heel rise. It highlights the importance of heel rise as a mechanism for controlling the downward movement of the centre of mass in late single support. It may help explain the observation of Williams et al. [43] that children with cerebral palsy, who have compromised plantarflexion function, often exhibit a low vertical component of the ground reaction in this phase.

Despite giving reasonable agreement with kinematics (Figure 3) Models 3 and 4 both require excessive moments at the hip, knee, and ankle (Figure 4) and this is only rectified when the contralateral limb is added in Model 5 (note that its mass was incorporated within the HAT segment in the earlier models). This indicates the influence of the dynamics of the contralateral limb on those of the stance limb. The acceleration of the contralateral limb in early swing and deceleration in late swing affect the requirements of the stance phase muscles at the corresponding times in single support. This is supported by data from Anderson and Pandy [8] which show substantial contributions to the vertical component of the ground reaction from muscles of the contralateral limb.

It is interesting that whilst Models 4 and 5 both allow heel rise, this does not occur until much later in the gait cycle than suggested by the experimental data (Figure 3) and all three models with an ankle joint show more dorsiflexion in late stance. Whilst no explanation for this could be found similarly excessive dorsiflexion was reported by Anderson and Pandy [44] and an even later heel rise [8].

Matches to the kinematic data are generally better earlier in the simulation (Figure 3). This is probably because the forward dynamics will lead to deviations here being propagated throughout the rest of the simulation having a relatively greater contribution to the cost functions than equivalent deviations later in the gait cycle. Apparent instabilities in the moments (Figure 4) may be attributable to similar effects. It may be that the cost function should weight kinematic deviations more heavily as the simulation progresses to account for this.

There are clearly still limitations with this model. One is that even the most complex model gives a simulation in which the ground reaction appears to underestimate the experimentally determined ground reaction force in early single support. The data compares quite well, however, with that of previous studies [8, 28] which show a less good match with the force data despite being considerably more complex. Further work extending the approach to a full gait cycle will allow incorporation of continuity constraints to ensure that the average force over the gait cycle under both feet is equal to bodyweight.

In summary the simulation of the single support phase of walking through Model 5 shows substantial agreement with experimental data in terms of the characteristics of the ground reaction force, joint angles, and moments. There are excessive dorsiflexion and late heel rise which have not been explained but are consistent with previous work. The joint moments appear a little erratic later on in the simulation which is because the cost function used is relatively insensitive to such variability. The consideration of a sequence of simulations of increasing complexity has given valuable insights into the mechanisms by which we walk, which are not appreciable from previous forward dynamic simulations using a single model.

## Figures and Tables

**Table 1:** The RMS errors from the experimental mean values for all models.

	Model 1	Model 2	Model 3	Model 4	Model 5	
					Stance	Swing
Segment angles (°)						
Foot	N/A	N/A	N/A	5.62	4.65	0.40
Tibia	N/A	6.67	2.36	1.84	1.00	0.93
Femur/total leg	0.27	4.18	0.93	1.64	2.14	0.63
HAT	0.49	0.31	0.06	0.02	0.02	
Joint moments (Nm)						
Ankle	N/A	N/A	23.63	21.41	10.06	1.07
Knee	N/A	48.11	26.52	23.50	5.63	8.07
Hip	0.00	34.8	24.24	24.84	31.16	15.63
GRF (%BW)						
*y*	31.94	71.91	20.32	8.91	9.45	
*x*	10.33	22.89	6.38	1.28	0.64	
